# Alterations of cell cycle control proteins SHP-1/2, p16, CDK4 and cyclin D1 in radioresistant nasopharyngeal carcinoma cells

**DOI:** 10.3892/mmr.2014.2463

**Published:** 2014-08-07

**Authors:** GANG PENG, RU-BO CAO, YUE-HUA LI, ZHEN-WEI ZOU, JING HUANG, QIAN DING

**Affiliations:** Department of Head and Neck Cancer, Cancer Center of Union Hospital, Tongji Medical College, Huazhong University of Science and Technology, Wuhan, Hubei 430022, P.R. China

**Keywords:** nasopharyngeal carcinoma, SHP-1, radiosensitivity, cell cycle, cyclin D1, p16

## Abstract

The primary treatment for nasopharyngeal carcinoma (NPC) is radiotherapy, with or without concurrent chemotherapy. However, resistance to radiotherapy is not uncommon. The aim of the present study was to establish a radioresistant NPC cell line to study the molecular mechanisms of radioresistance by measuring the expression of cell cycle control proteins src homology 2 domain-containing phosphatase (SHP)-1/2, p16, CDK4 and cyclin D1. Human nasopharyngeal carcinoma CNE-2 cells were cultured, divided into two groups (CNE-2S1 and CNE-2S2) and irradiated with a dose of 6 Gy x5 or 2 Gy x15, respectively. The cells were subcultured between doses of irradiation. The surviving sublines (CNE-2S1 and CNE-2S2 clones) were then passaged for three months and their radiosensitivity was determined. The cell cycle distribution and protein expression of SHP-1/2, p16, CDK4 and cyclin D1 in parental and progenitor cell lines were measured. Small interfering (si)RNA-mediated knockdown of SHP-1 and SHP-2 in the NPC cells was used to further examine their roles in radiosensitivity and cell cycle distribution. CNE-2S1, a radio-resistant cell line, had a significantly higher percentage of cells in S phase and a lower percentage of cells in G1 phase, enhanced expression levels of SHP-1, CDK4 and cyclin D1, and reduced expression of p16, respectively, as compared with the parent cells. Stable suppression of SHP-1 mRNA in CNE-2 cells resulted in increased radiosensitivity compared with the parental cells, a decrease in the number of cells in S phase and an increase in the expression of p16. The results suggested that the SHP-1/p16/cyclin D1/CDK4 pathway may have a role in regulating radiosensitivity and cell cycle distribution in nasopharyngeal cells.

## Introduction

Nasopharyngeal carcinoma (NPC), a malignant tumor derived from the epithelial cells of the nasopharynx, is associated with high rates of metastasis and a poor prognosis (1). NPC is particularly common in Guangdong and Guangxi in southern China, in Southeast Asia, Alaska and Greenland with a reported incidence as high as 50 cases/100,000 individuals (2). NPC occurs at an anatomical site which is poorly accessible to surgeons, and at first diagnosis >50% of patients have locally advanced, non-metastatic stage III or IV NPC (3,4). NPC is sensitive to ionizing radiation, thus radiotherapy, with or without concurrent chemotherapy, is the currently accepted standard of care (3–6). Survival is correlated with the disease stage at diagnosis with a five-year overall survival (OS) of 90% for stage I disease and 58% for stage IV disease (4,7). Despite the efficacy of radiotherapy, radioresistance remains a severe obstacle to effective treatment in numerous cases (6–8).

The presence of tumor cell heterogeneity is considered to result in significant differences in the intrinsic radiosensitivity of NPC cells, and the survival of radioresistant sublines following radiotherapy is the major cause of local recurrence and metastasis (8). Clinically relevant doses of radiation have been demonstrated to activate multiple signaling pathways, processes which are considered to depend on the expression of specific growth factor receptors, transcription factors, autocrine factors, RAS mutation and (phosphatase and tensin homolog) PTEN expression within the cell (9).

Recent studies have suggested that tyrosine phosphorylation and dephosphorylation, mediated by protein tyrosine kinases (PTK) and protein tyrosine phosphatases (PTP), have an important role in the regulation of these complex pathways (10–13). The src homology 2 domain-containing phosphatase (SHP) is a nuclear receptor with tumor-suppression activity and has been demonstrated to be downregulated in a number of cancer types (14). SHP-1 and SHP-2, src homology 2 (SH2) domain-containing phosphatases, share 55% sequence homology and a similar molecular structure (15). The SH2 domains of SHP-1 have recently been revealed to downregulate the activity of a number of kinases to negatively regulate signal transduction and to inhibit cell proliferation (14,16–21). Activated SHP-1 has been demonstrated to catalyze tyrosine dephosphorylation of the Janus kinases (JAKs), src and c-fms, leading to the reduction or loss of kinase activity and the inhibition of cell proliferation (14,16–19,22–26). SHP-1 has been reported to be a negative regulator of angiogenesis (27) and to be upregulated in breast cancer (28). Although SHP-1 was demonstrated to be a negative regulator of proliferation, knockdown of SHP-1 resulted in CDK6 downregulation and G1/S cell cycle arrest in prostate cancer cells (20,29). Several studies have identified that SHP-2 has a role in DNA damage-induced apoptosis and in the regulation of the DNA damage G2/M checkpoint (30,31). Furthermore, increased expression of SHP-2 has been found in gastric (32) and cervical cancer (33).

Retinoblastoma protein (pRb) inhibits the progression of cells into S phase and is regulated via pRb phosphorylation by the cyclin D1/cyclin-dependent kinase 4 (CDK4) complex (34). Tumor suppressor gene p16 has been demonstrated to negatively regulate cyclin D1/CDK4 complex phosphorylation activity and the cyclin D1/p16/Rb pathway appears to be altered in a number of malignancies (34). Hwang *et al* (35) reported that 89% of NPC tumors exhibited at least one alteration in the D1/p16/Rb pathway. Similarly, Gulley *et al* (36) found that p16 was not detectable in 64% of NPC cases.

The aim of the present study was to establish a radioresistant NPC cell line to study the molecular mechanism of radioresistance by measuring the expression of cell cycle control proteins SHP-1/2, p16, CDk4 and cyclin D1. The results may provide useful information for future improvements of radiotherapeutic strategies.

## Materials and methods

### Establishment of radioresistant nasopharyngeal carcinoma cell sublines

Human nasopharyngeal carcinoma CNE-2 cells were obtained from the Central Cancer Laboratory, Affiliated Union Hospital of Tongji Medical College, Huazhong University of Science and Technology (Wuhan, Hubei, China). The cells were cultured in RPMI-1640 (Gibco-BRL, Invitrogen Life Technologies, Carlsbad, CA, USA) supplemented with 10% fetal bovine serum (Hangzhou Evergreen Company, Hangzhou, China) at 37°C under 5% CO_2_.

Exponentially growing CNE-2 cells were divided into two groups (CNE-2S1 and CNE-2S2) and irradiated with a dose of 6 Gy x5 or 2 Gy x15, respectively. Irradiation was performed with 6 MV X-rays generated by a Siemens Primus H high-energy linear accelerator (Munich, Germany) as previously described (37). The length of the irradiation intervals were dependant on the MUs of LINAC delivered. There was a 7–9 day and 2–3 day break in between the 6 Gy x5 and 2 Gy x15 doses, respectively. The radiation field was 10×10 cm, the distance from the source to target was 100 cm and the absorbed dose rate was 200 cGy/min. The cells were subcultured between the doses of irradiation. The surviving sublines (CNE-2S1 and CNE-2S2 clones) were then passaged for three months and their radiosensitivity was determined.

### Construction of pGCsi-RNAi vectors

SHP-1 and SHP-2 RNAi target sequences were designed based on the NM_080549.3 and NM_002831.5 sequences obtained from the National Center for Biotechnology Information [NCBI; National Institutes of Health (NIH), Bethesda, MD, USA] database using online design software (http://rnaidesigner.invitrogen.com/rnaiexpress/). The target sequences are summarized in Table I. The negative control, p small interfering (si)RNA-NC, was not homologous to the target genes. CNE-2 cells were transiently transfected with the six different pGCsi-RNA plasmids or psiRNA-NC using Lipofectamine 2000 (Invitrogen Life Technologies, Carlsbad, CA, USA) according to the manufacturer’s instructions. Quantitative polymerase chain reaction (qPCR) and western blot analysis were performed to evaluate the interference efficiency 48 h following transfection.

### Detection of mRNA transcription using qPCR

Total RNA was extracted from transiently-transfected CNE-2 cells using TRIzol (Invitrogen Life Technologies) according to the manufacturer’s instructions. Total RNA (1 μg) was reversely transcribed using an oligo dT primer and moloney murine leukemia virus reverse transcriptase (Invitrogen Life Technologies) according to the manufacturer’s instructions. The cDNA product was PCR-amplified using SHP-1/2 primers (Table II). The cycling conditions were as follows: 35 cycles of denaturation at 95°C, annealing at 57°C and elongation at 72°C. GAPDH was used as an internal control. The amplified products were analyzed on 1% agarose gels.

### Western blot analysis

Total protein was extracted from transiently transfected CNE-2 cells using a lysis buffer (20 mM Tris [pH 7.5], 150 mM NaCl, 1% Triton X-100, sodium pyrophosphate, β-glycerophosphate, EDTA, Na_3_VO_4_ and leupeptin; Wuhan Biyuntian Biotechnology Research Institute, Shanghai, China) and quantified using a bicinchoninic acid kit (Biyuntian Biotechnology Research Institute, Shanghai, China). Equal amounts of protein were separated by SDS-PAGE and transferred to polyvinylidene fluoride membranes (Millipore, Billerica, MA, USA). The membranes were blocked with normal goat serum at 37°C for 1 h and were then incubated with a 1:300 dilution of primary rabbit anti-human SHP-1, p16, CDK4 or cyclin D1 monoclonal antibodies (Cell Signaling Technology, Inc., Danvers, MA, USA) at 4°C overnight. The membranes were extensively washed and incubated with a 1:2,000 dilution of horseradish peroxidase-conjugated goat anti-rabbit secondary antibody (Beijing Zhongshan Golden Bridge Company, Beijing, China) at 37°C for 1 h. The protein bands were visualized using an enhanced chemiluminescence kit (Pierce Biotechnology, Inc., Rockford, IL, USA) and visualized using a UV transilluminator (Uvitec Limited, Avebury House, Cambridge, UK). Image J 1.43b software (NIH, Bethesda, MD, USA) was used to scan the protein bands and to measure the optical density values. The ratio of the target band/internal reference GAPDH was calculated to determine the relative protein expression.

### Construction and identification of CNE-2 cell lines stably transfected with interference plasmids

CNE-2 cells were transfected with pGCsi-RNAi vectors for 24 h, passaged (1:10) and replated. The stably transfected cells were selected using 600 μg/ml G418 (Gibco-BRL) for 14 days. Positive clones were obtained and screened by qPCR and western blotting for transfection efficiency. The cells with high transfection efficiency were amplified and maintained in the presence of 300 μg/ml G418. The CNE-2 cells stably transfected with the siRNA inhibiting SHP-1 expression were named CNE-2S^*^, and CNE-2 cells stably transfected with the siRNA inhibiting SHP-2 expression were named CNE-2S^#^.

### Clonogenic survival assay

Single-cell suspensions of parental CNE-2, CNE-2S1, CNE-2S2, CNE-2S^*^ and CNE-2S^#^ cells were plated in six-well culture plates and irradiated with 0, 200, 400, 600, 800 and 1,000 cGy. Following irradiation, the cells were cultured for two weeks, fixed with absolute ethanol containing 1% methyl violet for 20 min and the number of surviving colonies (defined as a colony with >50 cells) were counted. The plating efficiency (PE) and the cell survival fraction (SF) were calculated as follows: PE = (number of colonies in the control group/number of inoculated cells) × 100% and SF = (number of colonies in the experimental group/number of inoculated cells) × PE. The cell survival curves were plotted with Sigma Plot 2001 software using the multi-target, single-hit model S = l-(1-e^−D/D0^)^N^. Radiobiological parameters, including the average lethal dose (D_0_), quasi-threshold dose (D_q_) and the extrapolation number (N), were also calculated (38).

### Cell cycle detection with flow cytometry

Single cell suspensions of irradiated CNE-2S1 and CNE-2S2 cells were added to pre-chilled 75% ethanol and fixed at −20°C overnight. The ethanol was then discarded and the cells were rinsed with phosphate-buffered saline and resuspended. The samples were digested with RNAase and propidium iodide (PI; Sigma, St. Louis, MO, USA) was added to achieve a final concentration of 60 μg/ml. The samples were incubated in the dark for 30 min and subjected to flow cytometry using FACScan (Becton Dickinson, San Jose, CA, USA) using blue light (488 nm) for excitation. Fluorescence was measured at 530±20 nm (green, fluorescein isothiocyanate) and >620 nm (red, PI) (39). The experiment was repeated three times and the mean was calculated.

### Statistical analysis

Data are presented as the mean ± standard deviation. Differences between the three groups were assessed by analysis of variance for continuous variables. The Bonferroni method was used for adjustment of type I errors for multiple comparisons. All statistical assessments were evaluated at a two-sided α-level of 0.05. Statistical analyses were performed using SAS 9.2 statistics software (SAS Institute Inc., Cary, NC, USA).

## Results

### Radiosensitivity of CNE-2S1 and CNE-2S2 cells

**Figure 1:**
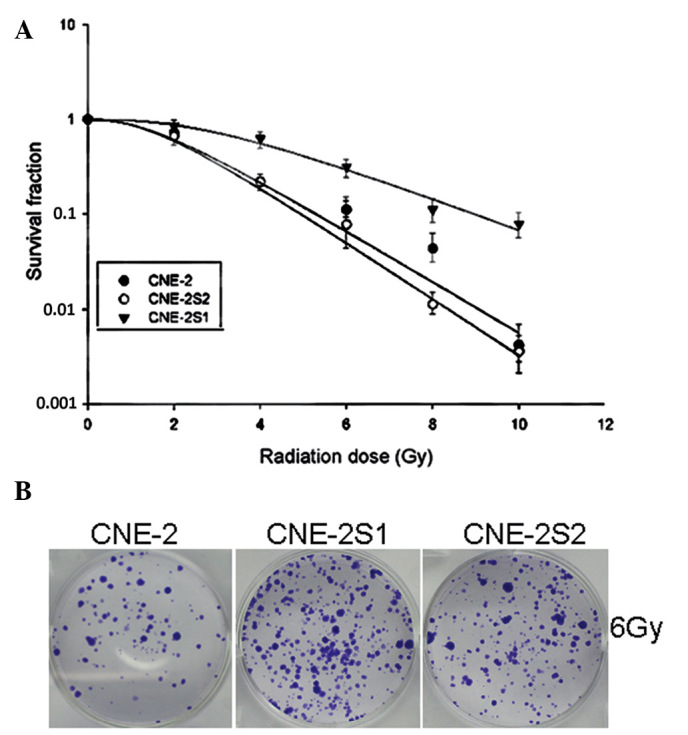
(A) Cell survival curves of CNE-2, CNE-2S1 and CNE-2S2 cells plotted by the multi-target single-hit model. (B) Representative images of the clonogenic survival assay of these three lines. Error bars represent the mean ± standard deviation.

The fractions of CNE-2, CNE-2S1 and CNE-2S2 cells that survived irradiation are revealed in Fig. 1A. The results demonstrated that irradiation killed the cells logarithmically and that CNE-2S1 cells had a higher radioresistance compared with the parental or CNE-2S2 cells. Notably, CNE-2S1 cells had higher D_0_, D_q_ and N values compared with the parental CNE-2 cells, indicating higher radioresistance (Table III). By contrast, D_0_, D_q_ and N values were similar between the CNE-2S2 and parental cells, indicating no difference in radiosensitivity.

CNE-2, CNE-2S1 and CNE-2S2 cells were cultured for three months, and analysis of the cell survival curves as well as the radiosensitivity parameters D_0_, D_q_ and N indicated that CNE-2S1 cells had higher radioresistance compared with the parental and CNE-2S2 cells following three months of culture (Table IV).

### Cell cycle analysis of CNE-2, CNE-2S1 and CNE-2S2 cells

**Figure 2:**
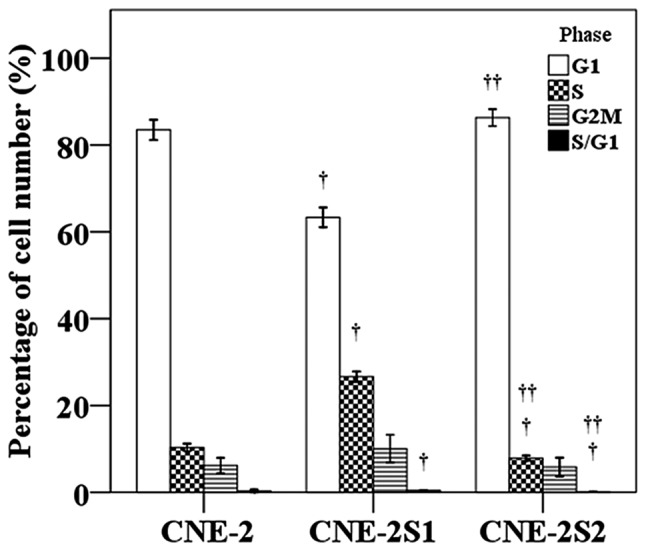
Cell cycle analysis of CNE-2, CNE-2S1 and CNE-2S2 cells. ^†^Significant difference as compared with the CNE-2 group. ^††^Significant difference as compared with the CNE-2S1 group. Error bars represent the mean ± standard deviation.

Irradiated CNE-2S1 cells had a significantly lower percentage of cells in G1 phase and a significantly higher percentage of cells in S phase compared with irradiated CNE-2 and CNE-2S2 cells (Fig. 2). In the CNE-2 group, 83.5±2.3% of cells were in G1 phase, 10.3±0.7% cells were in S phase and 6.2±1.8% were in G2-M phase. The CNE-2S2 group demonstrated a similar profile with 86.3±2.0% cells in G1 phase, 7.9±0.6% in S phase and 5.8±2.2% in G2-M phase. However, in the CNE-2S1 group, 63.3±2.8% of the cells were in G1 phase, 26.6±1.2% were in S phase and 10±3.2% were in G2-M phase. The average S/G1 ratios in the CNE-2, CNE-2S1 and CNE-2S2 groups were 0.12, 0.42 and 0.09, respectively.

### SHP-1/2, p16, CDK4 and cyclin D1 expression in CNE-2, CNE-2S1 and CNE-2S2 cells

**Figure 3:**
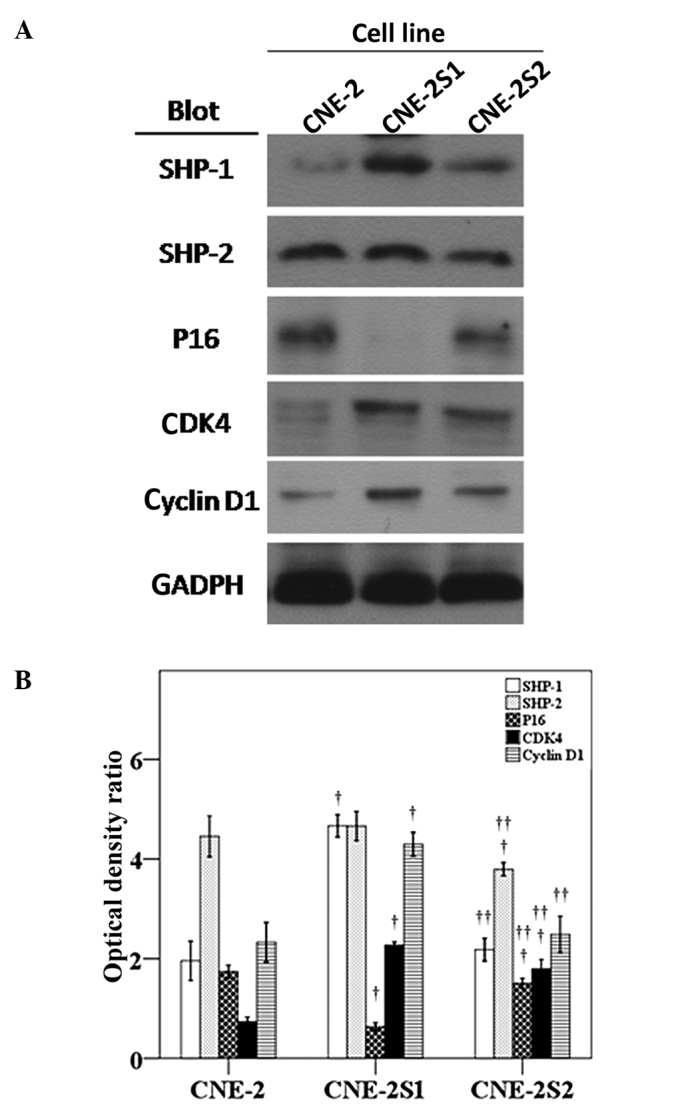
Comparison of SHP-1/2, p16, CDK4 and cyclin D1 expression in CNE-2, CNE-2S1 and CNE-2S2 cells. (A) Western blotting image. (B) Quantification of western blotting data. ^†^Significant difference as compared with the CNE-2 group; ^††^significant difference as compared with the CNE-2S1 group. Error bars represent the mean ± standard deviation. SHP-1/2, src homology 2 domain-containing phosphatase-1/2; CDK16, cyclin-dependent kinase 16.

There was a significant upregulation of SHP-1, CDK4 and cyclin D1 and a significant downregulation of p16 in CNE-2S1 cells as compared with CNE-2 cells (Fig. 3). There was significant downregulation of SHP-2 and p16, and an upregulation of CDK4 in CNE-2S2 cells compared with the CNE-2 cells. As compared with the CNE-2S1 cells, in CNE-2S2 there was a significant upregulation of p16 and a significant downregulation of all other proteins studied.

### Survival of CNE-2S^*^ and CNE-2S^#^ cells following different radiation doses

**Figure 4:**
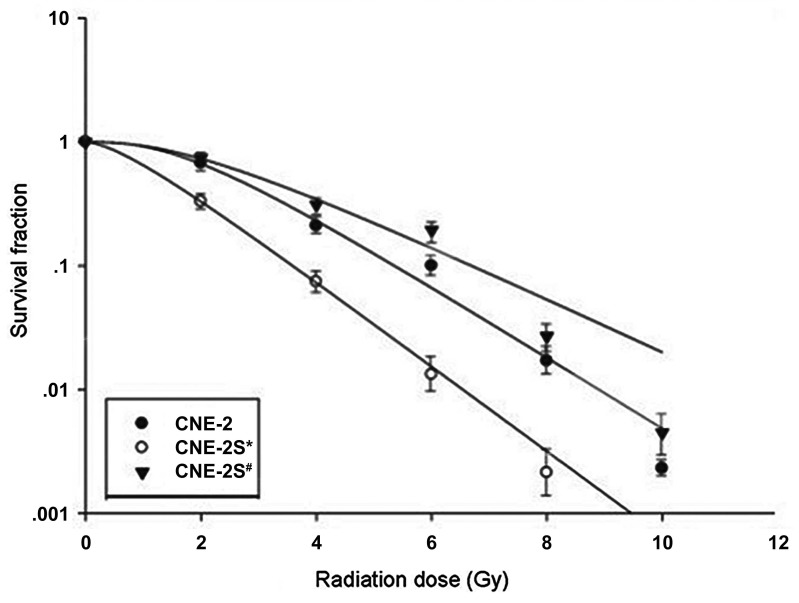
Cell survival curves of CNE-2, of CNE-2S^#^ and CNE-2S^*^ cells subjected to different radiation doses. Error bars represent the mean ± standard deviation. CNE-2S^*^, SHP-1-silenced CNE-2 cells; CNE-2S^#^, SHP-2-silenced CNE-2 cells; SHP-1/2, src homology 2 domain-containing phosphatase-1/2.

The survival curves of the CNE-2S^*^ and CNE-2S^#^ cell lines are demonstrated in Fig. 4, and the radiosensitivity parameters are summarized in Table V. In all of the cell lines, the radiation killed the cells in a logarithmic dose-dependent manner. The CNE-2S^*^ cells had significantly lower D_0_, D_q_ and N values and a narrower shoulder area under the cell survival curve compared with the CNE-2 cells, suggesting that these cells were more radiosensitive compared with CNE-2 cells.

### Cell cycle analysis of CNE-2, CNE-2S^#^ and CNE-2S^*^ cells

**Figure 5:**
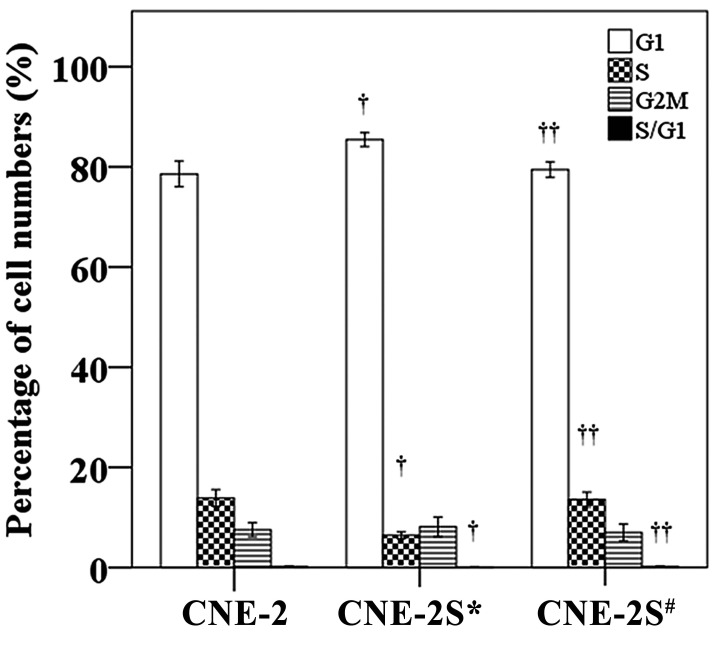
Cell cycle analysis of CNE-2, CNE-2^*^ and CNE-2^#^ cells. ^†^Significant difference as compared with the CNE-2 group; ^††^significant difference as compared with the CNE-2S^*^ group. Error bars represent the mean ± standard deviation. CNE-2S^*^, SHP-1-silenced CNE-2 cells; CNE-2S^#^, SHP-2-silenced CNE-2 cells; SHP-1/2, src homology 2 domain-containing phosphatase-1/2.

The results of the flow cytometric cell cycle analysis of the CNE-2, CNE-2S^#^ and CNE-2S^*^ cells are shown in Fig. 5. In the CNE-2 group, 78.6±2.6, 13.9±1.7 and 7.5±1.4% of the cells were in G1, S, and G2-M phase, respectively. The cell cycle profile of the CNE-2S^#^ group revealed 79.4±1.6, 13.6±1.5 and 7.0±1.7% of cells in G1, S and G2-M phase, respectively; however there was no significant difference from the DNE-2 group. The CNE-2S^*^ group exhibited a significant difference in the percentage of G1, S and S/G1 cells compared with the CNE-2S^#^ and CNE-2 groups, with 85.4±1.4, 6.4±0.7 and 10.0±2.0% cells in G1, S and G2-M phase, respectively. There was no significant difference in the percentage of G2-M cells between the three groups. The average percentage of S/G1 phase cells in the CNE-2, CNE-2S^*^ and CNE-2S^#^ groups was 0.2, 0.1 and 0.2, respectively.

### SHP-1/2, p16, CDK4 and cyclin D1 expression in CNE-2, CNE-2S^*^ and CNE-2S^#^ cells

**Figure 6:**
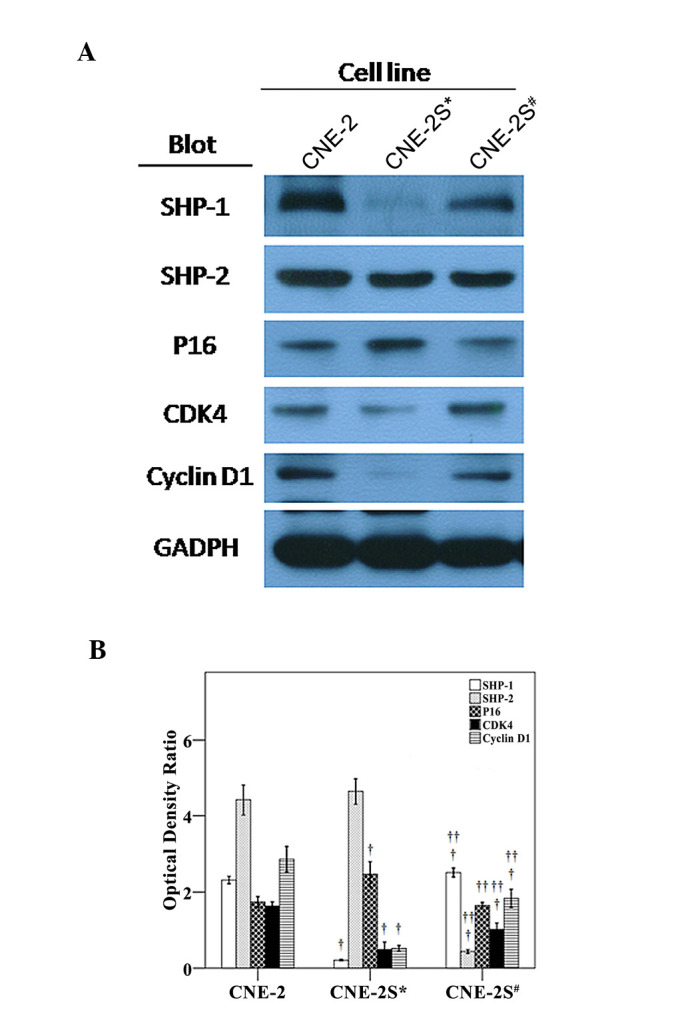
Comparison of SHP-1/2, p16, CDK4 and cyclin D1 expression in CNE-2, CNE-2S^*^ and CNE-2S^#^ cells. (A) Western blotting image. (B) Quantification of western blotting data. ^†^Significant difference as compared with the CNE-2 group; ^††^significant difference as compared with the CNE-2S^#^ group. Error bars represent the mean ± standard deviation. SHP-1/2, src homology 2 domain-containing phosphatase-1/2; CDK16, cyclin-dependent kinase 16. CNE-2S^*^, SHP-1-silenced CNE-2 cells; CNE-2S^#^, SHP-2-silenced CNE-2 cells; SHP-1/2, src homology 2 domain-containing phosphatase-1/2.

The expression levels of SHP-1, CDK4 and cyclin D1 were significantly downregulated, while the expression of p16 was significantly upregulated in the CNE-2S^*^ cells compared with the CNE-2 cells (Fig. 6). SHP-2 and CDK4 were significantly downregulated in the CNE-2S^#^ cells compared with the CNE-2 cells. As compared with the CNE-2S^*^ cells, the expression of SHP-1, CDK4 and cyclin D1 was significantly upregulated and the expression levels of SHP-2 and p16 were downregulated in the CNE-2S^#^ cells.

### SHP-1 in radioresistant lung cancer cell lines A549S1 and A549S2

**Figure 7:**
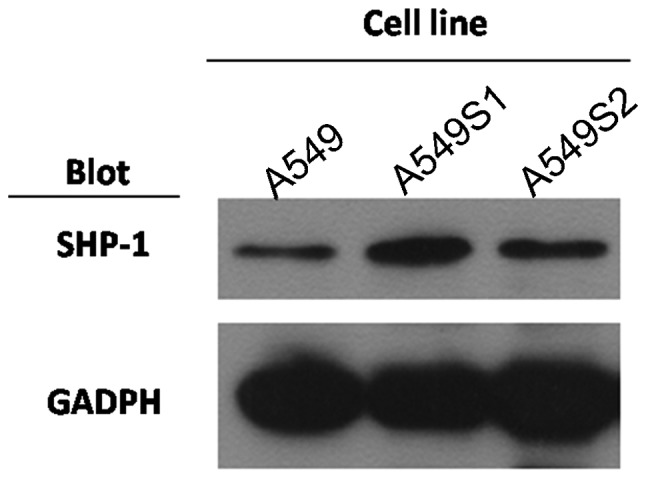
SHP-1 expression in radioresistant lung cancer cell lines. SHP-1, src homology 2 domain-containing phosphatase-1/2.

Upregulation of SHP-1 was not only detected in radioresistant nasopharyngeal carcinoma cells; radioresistance was also established in the lung cancer cell lines A549S1 and A549S2. Compared with the parent line A549, SHP-1 expression, as determined by western blotting, in these two radioresistant lines was similarly increased as observed in the CNE-2S1 cells (Fig. 7).

## Discussion

The results of the present study demonstrated that CNE-2S1 cells were significantly more radioresistant than CNE-2S2 cells and parental cells, had a significantly higher percentage of cells in S phase and a significantly lower percentage of cells in G1 phase as compared with CNE-2S2 cells. Significantly higher levels of SHP-1, CDK4 and cyclin D1 protein and significantly lower levels of p16 were found in the CNE-2S1 compared with the CNE-2S2 cells. Stable suppression of SHP-1 mRNA in CNE-2 cells resulted in increased radiosensitivity compared with the parental cells, a decrease in the number of cells in the S phase and an increase in the expression of p16. Taken together, the results suggested that the SHP-1/p16/cyclin D1/CDK4 pathway may have a role in regulating radiosensitivity and cell cycle distribution in nasopharyngeal cells.

These data demonstrated that the large split-dose irradiation induced the formation of more radioresistant NPC cells compared with the conventional fractionation method. Fractionated radiotherapy has been demonstrated to lead to radioresistance via a number of different mechanisms, including i) selection of an intrinsic radioresistant phenotype from a heterogenous population; ii) induction of mutations leading to radioresistance and iii) alterations in the tumor microenvironment (40). Radiosensitivity has been reported to be affected by cellular hypoxia, efficiency of the DNA repair mechanisms following radiation-induced DNA damage, the number of dividing cells and the cell cycle distribution (41,42). Abrogation of the G2/M checkpoint has been reported to potentiate radiation-induced cell death and the checkpoint kinase 1 inhibitor Go6976 was demonstrated to enhance the sensitivity of NPC cells to radiotherapy (43). Acquired radioresistance was also revealed to be associated with cyclin D1 overexpression (40). In general, cells in S phase are radioresistant, cells in G0/1 phase are relatively radiosensitive and cells in the G2-M phase are most sensitive to radiation (38). The present data demonstrated a significantly higher proportion of S phase cells and a significantly lower proportion of G1 phase cells in the CNE-2S1 group compared with the parental cells, while there was no difference in the percentage of G2/M cells. Of note, there were no significant changes in the proportions of cells in the various cell cycle phases in the CNE-2S2 cells. Based on these data, it was hypothesized that the dysregulation of the cell cycle may be an important mechanism driving radioresistance in NPC cells.

SHP-1 is expressed in hematopoietic cells, as well as other cell types, and in malignant cells, most notably in malignant epithelial cells (21). SHP-1 regulates cell proliferation by catalyzing tyrosine dephosphorylation, leading to the reduction or loss of kinase activity and by regulating proteins important in the cell-cycle, including CDK2, p27 and cyclin D1 (21). Although SHP-1 has been demonstrated to be an inhibitor of cell proliferation, knockdown of SHP-1 was recently reported to downregulate CDK6 and inhibit G1/S progression in prostate cancer cells (20). The present results, which demonstrated that SHP-1 knockdown resulted in a G1/ S block accompanied by a significant increase in radiosensitivity, are consistent with those of the aforementioned study. SHP-1 has previously been suggested to interact with PI3K to increase the protein stability of p27 and to modulate cell cycle events (20). In addition, Seo *et al* (27) demonstrated that SHP-1 mediates the anti-proliferative activity of the tissue inhibitor of metalloproteinase (TIMP)-2 in human microvascular endothelial cells.

The present study investigated the association between SHP-1 and p16, as p16 has previously been demonstrated to be silenced in the vast majority of NPC patients (35,36). In addition, low p16 expression correlated with poor outcome and adenovirus-mediated p16 gene therapy inhibited tumor formation in a mouse model of NPC (44). The data of the present study are consistent with these results and demonstrated a significant downregulation of p16 in CNE-2S1 cells, which was reversed in the CNE-2S^*^ cells, where SHP-1 expression was silenced.

Areas of future study include the correlation of SHP-1 and radiation-induced signaling through pro-survival pathways (e.g., epidermal growth factor receptor; PI3K/Akt), as well as the correlation with the expression of radiation-activated transcription factor (activator protein 1 and nuclear factor κB), and the expression of p21 and p27kip1 in the NPC cell lines studied (45–47).

In conclusion, the results of the present study demonstrated that SHP-1 has a role in the radioresistance of NPC cells, possibly via the regulation of the cell cycle. Targeting specific signaling pathways to modulate radiosensitivity may be valuable in the development of novel therapeutic strategies to treat NPC.

## Figures and Tables

**Table I:** SHP-1 and SHP-2 RNAi target sequences.

A, SHP-1 siRNA target sequences		

Plasmid	siRNA sequence	Start site
pGCsi-RNA 1	5′-TCCCGACAACACAATACCAGATAAATTC	
	AAGAGATTTATCTGGTATTGTGTTGTCTTT-3′	1907
pGCsi-RNA 2	5′-TCCCGTCCCATTACTACTGTTCCAATTC	
	AAGAGATTGGAACAGTAGTAATGGGACTT-3′	774
pGCsi-RNA 3	5′-TCCCAATCTCTATGCAACTCAAGGCTTC	
	AAGAGAGCCTTGAGTTGCATAGAGATTTT-3′	970
pGCsi-RNA NC	5′-TCCCTTCTCCGAACGTGTCACGTTTC	
	AGAGAACGTGACACGTTCGGAGAATT-3′	

B, SHP-2 siRNA target sequences		

Plasmid	siRNA sequence	Start site

pGCsi-RNA 4	5′-TCCCGACCCTTATCGTACGATCTAATAAATTC	
	AAGAGATTCTACTATCTTACTTATTATCTATTT-3′	1907
pGCsi-RNA 5	5′-TCCCCTTATCTATCTATCTGATGGATTTC	
	AAGAGAATCGATCGATCGATCACTGATCGTT-3′	774
pGCsi-RNA 6	5′-TCCCATCTATGCTCGCGCTAGCTCGATGTTC	
	AAGAGAATTCTATCTATATATCTGGTATGTT-3′	970
pGCsi-RNA NC	5′-TCCCTTCTCCGAACGTGTCACGTTTC	
	AGAGAACGTGACACGTTCGGAGAATT-3′	

SHP-1/2, src homology 2 domain-containing phosphatase-1/2; siRNA, small interfering RNA; NC, negative control.

**Table II:** SHP-1/2 polymerase chain reaction primers.

Target gene	Primers	Annealing temperature (°C)	Length of product (bp)
SHP-1	F: 5′-TTGTAGCACTCCGAATGGTT-3′ R: 5′-CTTCTGCCTGGTCTTCTCCT-3′	56.6	185
SHP-2	F: 5′-TCCAGGACTGCAATGCTTAC-3′ R: 5′-CCTAATTCGGATCGTAGCTAATG-3′	58.6	174

SHP-1/2, src homology 2 domain-containing phosphatase-1/2; bp, base pairs; F, forward; R, reverse.

**Table III:** Radiosensitivity parameters of the cell lines.

Parameter	CNE-2	CNE-2S1	CNE-2S2	P-value
D0	1.71±0.03	2.07±0.07^b^	1.62±0.05^c^	<0.001^a^
Dq	1.49±0.06	2.01±0.08^b^	1.46±0.03^c^	<0.001^a^
N	2.87±0.05	4.43±0.14^b^	2.45±0.11^b^,^c^	<0.001^a^

Continuous data presented as the mean ± standard deviation and compared between different groups by one-way analysis of variance with Bonferroni post-hoc test for type I error adjustment.

a Significant difference among the three groups;

b significant difference as compared with the CNE-2 group;

c significant difference as compared with the CNE-2S1 group.

D0, average lethal dose; Dq, quasi-threshold dose; N, extrapolation number.

**Table IV:** Radiosensitivity parameters of the cell lines following three months of culture.

Parameter	CNE-2	CNE-2S1	CNE-2S2	P-value
D0	1.42±0.05	2.13±0.04^b^	1.39±0.07^c^	<0.001^a^
Dq	1.57±0.06	2.41±0.07^b^	1.69±0.08^c^	<0.001^a^
N	2.38±0.11	4.35±0.13^b^	2.47±0.05^c^	<0.001^a^

Continuous data presented as the mean ± standard deviation and compared between different groups by one-way analysis of variance with Bonferroni post hoc test for type I error adjustment.

a Significant difference among the three groups;

b significant difference as compared with the CNE-2 group;

c significant difference as compared with the CNE-2S1 group.

CD0, average lethal dose; Dq, quasi-threshold dose; N, extrapolation number.

**Table V:** Radiosensitivity parameters of CNE-2S^*^ and CNE-2S^#^ cells after different radiation doses.

Parameter	CNE-2	CNE-2S^*^	CNE-2S^#^	P-value
D_0_	1.64±0.08	1.23±0.04^b^	1.83±0.06^b^,^c^	<0.001^a^
D_q_	1.25±0.03	0.93±0.05^b^	1.35±0.05^b^,^c^	<0.001^a^
N	2.35±0.09	1.89±0.06^b^	2.49±0.11^c^	<0.001^a^

Continuous data presented as the mean ± standard deviation and compared between different groups by one-way analysis of variance with Bonferroni post-hoc test for type I error adjustment.

a Significant difference among the three groups;

b significant difference as compared with the CNE-2 group;

c significant difference as compared with the CNE-2S^*^ group.

D_0_, average lethal dose; D_q_, quasi-threshold dose; N, extrapolation number. CNE-2S^*^, SHP-1-silenced CNE-2 cells; CNE-2S^#^, SHP-2-silenced CNE-2 cells; SHP-1/2, src homology 2 domain-containing phosphatase-1/2.

## Abbreviations

NPC: nasopharyngeal carcinoma
CRT: chemotherapy-radiotherapy
EGFR: epidermal growth factor receptor
MV: megavolt
PE: inoculation efficiency
SF: cell survival fraction
D0: average lethal dose
Dq: quasi-threshold dose
N: extrapolation number
PTK: protein tyrosine kinases
PTP: protein tyrosine phosphatases

## References

[1] Tulalamba W, Janvilisri T (2012). Nasopharyngeal carcinoma signaling pathway: an update on molecular biomarkers. Int J Cell Biol.

[2] Chang ET, Adami HO (2006). The enigmatic epidemiology of nasopharyngeal carcinoma. Cancer Epidemiol Biomarkers Prev.

[3] Lee AW, Sze WM, Au JS, Leung SF, Leung TW, Chua DT, Zee BC, Law SC, Teo PM, Tung SY, Kwong DL, Lau WH (2005). Treatment results for nasopharyngeal carcinoma in the modern era: the Hong Kong experience. Int J Radiat Oncol Biol Phys.

[4] Lee AW, Poon YF, Foo W, Law SC, Cheung FK, Chan DK, Tung SY, Thaw M, Ho JH (1992). Retrospective analysis of 5037 patients with nasopharyngeal carcinoma treated during 1976–1985 overall survival and patterns of failure. Int J Radiat Oncol Biol Phys.

[5] Langendijk JA, Leemans CR, Buter J, Berkhof J, Slotman BJ (2004). The additional value of chemotherapy to radiotherapy in locally advanced nasopharyngeal carcinoma: a meta-analysis of the published literature. J Clin Oncol.

[6] Lin JC, Jan JS, Hsu CY, Liang WM, Jiang RS, Wang WY (2003). Phase III study of concurrent chemoradiotherapy versus radiotherapy alone for advanced nasopharyngeal carcinoma: positive effect on overall and progression-free survival. J Clin Oncol.

[7] Wee J, Tan EH, Tai BC, Wong HB, Leong SS, Tan T, Chua ET, Yang E, Lee KM, Fong KW, Tan HS, Lee KS, Loong S, Sethi V, Chua EJ, Machin D (2005). Randomized trial of radiotherapy versus concurrent chemoradiotherapy followed by adjuvant chemotherapy in patients with American Joint Committee on Cancer/International Union against cancer stage III and IV nasopharyngeal cancer of the endemic variety. J Clin Oncol.

[8] Feng XP, Yi H, Li MY, Li XH, Yi B, Zhang PF, Li C, Peng F, Tang CE, Li JL, Chen ZC, Xiao ZQ (2010). Identification of biomarkers for predicting nasopharyngeal carcinoma response to radiotherapy by proteomics. Cancer Res.

[9] Dent P, Yacoub A, Contessa J, Caron R, Amorino G, Valerie K, Hagan MP, Grant S, Schmidt-Ullrich R (2003). Stress and radiation-induced activation of multiple intracellular signaling pathways. Radiat Res.

[10] Sung FL, Hui EP, Tao Q, Li H, Tsui NB, Dennis Lo YM, Ma BB, To KF, Harris AL, Chan AT (2007). Genome-wide expression analysis using microarray identified complex signaling pathways modulated by hypoxia in nasopharyngeal carcinoma. Cancer Lett.

[11] Morrison JA, Gulley ML, Pathmanathan R, Raab-Traub N (2004). Differential signaling pathways are activated in the Epstein-Barr virus-associated malignancies nasopharyngeal carcinoma and Hodgkin lymphoma. Cancer Res.

[12] Li L, Tao Q, Jin H, van Hasselt A, Poon FF, Wang X, Zeng MS, Jia WH, Zeng YX, Chan AT, Cao Y (2010). The tumor suppressor UCHL1 forms a complex with p53/MDM2/ARF to promote p53 signaling and is frequently silenced in nasopharyngeal carcinoma. Clin Cancer Res.

[13] Zeng ZY, Zhou YH, Zhang WL, Xiong W, Fan SQ, Li XL, Luo XM, Wu MH, Yang YX, Huang C, Cao L, Tang K, Qian J, Shen SR, Li GY (2007). Gene expression profiling of nasopharyngeal carcinoma reveals the abnormally regulated Wnt signaling pathway. Hum Pathol.

[14] Oka T, Yoshino T, Hayashi K, Ohara N, Nakanishi T, Yamaai Y, Hiraki A, Sogawa CA, Kondo E, Teramoto N, Takahashi K, Tsuchiyama J, Akagi T (2001). Reduction of hematopoietic cell-specific tyrosine phosphatase SHP-1 gene expression in natural killer cell lymphoma and various types of lymphomas/leukemias: combination analysis with cDNA expression array and tissue microarray. Am J Pathol.

[15] Neel BG, Gu H, Pao L (2003). The ‘Shp’ing news: SH2 domain-containing tyrosine phosphatases in cell signaling. Trends Biochem Sci.

[16] Mittal Y, Pavlova Y, Garcia-Marcos M, Ghosh P (2011). Src homology domain 2-containing protein-tyrosine phosphatase-1 (SHP-1) binds and dephosphorylates G(alpha)-interacting, vesicle-associated protein (GIV)/Girdin and attenuates the GIV-phosphatidylinositol 3-kinase (PI3K)-Akt signaling pathway. J Biol Chem.

[17] Beyaert R (2011). SHP works a double shift to control TLR signaling. Nat Immunol.

[18] Prasad S, Pandey MK, Yadav VR, Aggarwal BB (2011). Gambogic acid inhibits STAT3 phosphorylation through activation of protein tyrosine phosphatase SHP-1: potential role in proliferation and apoptosis. Cancer Prev Res (Phila).

[19] López-Ruiz P, Rodriguez-Ubreva J, Cariaga AE, Cortes MA, Colás B (2011). SHP-1 in cell-cycle regulation. Anticancer Agents Med Chem.

[20] Rodríguez-Ubreva FJ, Cariaga-Martinez AE, Cortés MA, Romero-De Pablos M, Ropero S, López-Ruiz P, Colás B (2010). Knockdown of protein tyrosine phosphatase SHP-1 inhibits G1/S progression in prostate cancer cells through the regulation of components of the cell-cycle machinery. Oncogene.

[21] López-Ruiz P, Rodriguez-Ubreva J, Cariaga AE, Cortes MA, Colás B (2011). SHP-1 in cell-cycle regulation. Anticancer Agents Med Chem.

[22] Yu Z, Maoui M, Zhao ZJ, Li Y, Shen SH (2006). SHP-1 dephosphorylates 3BP2 and potentially downregulates 3BP2-mediated T cell antigen receptor signaling. FEBS J.

[23] Zhang J, Shen B (2011). SHP limits TLR signaling, an inducible transcriptional corepressor. Cell Mol Immunol.

[24] Iype T, Sankarshanan M, Mauldin IS, Mullins DW, Lorenz U (2010). The protein tyrosine phosphatase SHP-1 modulates the suppressive activity of regulatory T cells. J Immunol.

[25] Biskup C, Böhmer A, Pusch R, Kelbauskas L, Gorshokov A, Majoul I, Lindenau J, Benndorf K, Böhmer FD (2004). Visualization of SHP-1-target interaction. J Cell Sci.

[26] Zhu Z, Oh SY, Cho YS, Zhang L, Kim YK, Zheng T (2010). Tyrosine phosphatase SHP-1 in allergic and anaphylactic inflammation. Immunol Res.

[27] Seo DW, Li H, Qu CK, Oh J, Kim YS, Diaz T, Wei B, Han JW, Stetler-Stevenson WG (2006). Shp-1 mediates the antiproliferative activity of tissue inhibitor of metalloproteinase-2 in human microvascular endothelial cells. J Biol Chem.

[28] Yip SS, Crew AJ, Gee JM, Hui R, Blamey RW, Robertson JF, Nicholson RI, Sutherland RL, Daly RJ (2000). Up-regulation of the protein tyrosine phosphatase SHP-1 in human breast cancer and correlation with GRB2 expression. Int J Cancer.

[29] Ketroussi F, Giuliani M, Bahri R, Azzarone B, Charpentier B, Durrbach A (2011). Lymphocyte cell-cycle inhibition by HLA-G is mediated by phosphatase SHP-2 and acts on the mTOR pathway. PLoS One.

[30] Yuan L, Yu WM, Qu CK (2003). DNA damage-induced G2/M checkpoint in SV40 large T antigen-immortalized embryonic fibroblast cells requires SHP-2 tyrosine phosphatase. J Biol Chem.

[31] Yuan L, Yu WM, Yuan Z, Haudenschild CC, Qu CK (2003). Role of SHP-2 tyrosine phosphatase in the DNA damage-induced cell death response. J Biol Chem.

[32] Jiang J, Jin MS, Kong F, Wang YP, Jia ZF, Cao DH, Ma HX, Suo J, Cao XY (2013). Increased expression of tyrosine phosphatase SHP-2 in *Helicobacter pylori*-infected gastric cancer. World J Gastroenterol.

[33] Meng F, Zhao X, Zhang S (2012). Expression and significance of SHP-2 in human papillomavirus infected cervical cancer. J Huazhong Univ Sci Technolog Med Sci.

[34] Hall M, Peters G (1996). Genetic alterations of cyclins, cyclin-dependent kinases and Cdk inhibitors in human cancer. Adv Cancer Res.

[35] Hwang CF, Cho CL, Huang CC, Wang JS, Shih YL, Su CY, Chang HW (2002). Loss of cyclin D1 and p16 expression correlates with local recurrence in nasopharyngeal carcinoma following radiotherapy. Ann Oncol.

[36] Gulley ML, Nicholls JM, Schneider BG, Amin MB, Ro JY, Geradts J (1998). Nasopharyngeal carcinomas frequently lack the p16/MTS1 tumor suppressor protein but consistently express the retinoblastoma gene product. Am J Pathol.

[37] Pearce AG, Segura TM, Rintala AC, Rintala-Maki ND, Lee H (2001). The generation and characterization of a radiation-resistant model system to study radioresistance in human breast cancer cells. Radiat Res.

[38] Hall EJ, Giaccia A (2011). Radiobiology for the Radiologist. Section I, Chapter 3: Cell survival curves.

[39] Larsen JK, Landberg G, Roos G (2001). Detection of proliferating cell nuclear antigen. Methods Cell Biol.

[40] Shimura T (2011). Acquired radioresistance of cancer and the AKT/GSK3β/cyclin D1 overexpression cycle. J Radiat Res.

[41] Deorukhkar A, Krishnan S (2010). Targeting inflammatory pathways for tumor radiosensitization. Biochem Pharmacol.

[42] Brizel DM, Sibley GS, Prosnitz LR, Scher RL, Dewhirst MW (1997). Tumor hypoxia adversely affects the prognosis of carcinoma of the head and neck. Int J Radiat Oncol Biol Phys.

[43] Feng Z, Xu S, Liu M, Zeng YX, Kang T (2010). Chk1 inhibitor Gö6976 enhances the sensitivity of nasopharyngeal carcinoma cells to radiotherapy and chemotherapy in vitro and in vivo. Cancer Lett.

[44] Li AA, Ng E, Shi W, Lee A, Chia M, Liu TJ, Huang D, O’Sullivan B, Gullane P, Liu FF (2004). Potential efficacy of p16 gene therapy for EBV-positive nasopharyngeal carcinoma. Int J Cancer.

[45] Gupta S, Ahmed MM (2004). A global perspective of radiation-induced signal transduction pathways in cancer therapeutics. Indian J Exp Biol.

[46] Storozhuk Y, Sanli T, Hopmans SN, Schultz C, Farrell T, Cutz JC, Steinberg GR, Wright J, Singh G, Tsakiridis T (2012). Chronic modulation of AMP-Kinase, Akt and mTOR pathways by ionizing radiation in human lung cancer xenografts. Radiat Oncol.

[47] Valerie K, Yacoub A, Hagan MP, Curiel DT, Fisher PB, Grant S, Dent P (2007). Radiation-induced cell signaling: inside-out and outside-in. Mol Cancer Ther.

